# THE HIGH COST OF SOCIOECONOMICS ON RACIAL DISPARITIES IN HEALTHY AGING

**DOI:** 10.1093/geroni/igz038.2193

**Published:** 2019-11-08

**Authors:** Nancy Chiles Shaffer

**Affiliations:** National Institute on Aging, Baltimore, Maryland, United States

## Abstract

Race-related differences in overall health across the age-span are well established; how differences manifest within a cohort selected for good functional status is unclear. Using data from Black and White older adults aged 70-79, in the Health, Aging and Body Composition (Health ABC) study, we created a healthy aging index (HAI) of mental health, fitness, lung capacity, bone mineral density, inflammation, and metabolic syndrome. We assessed if racial differences existed in HAI, the extent education, financial resources and stress attenuated any observed differences, and whether this varied by site (Memphis v. Pittsburgh). Blacks had lower HAI than whites, adjusted for age and site. Further adjustment for finances and education eliminated the effect of race in women and reduced the effect in men by 64%. A significant interaction between site and financial stress was observed. Future research should assess ways to reduce the harmful impact of low socioeconomic status on health.

